# Graphene-oxide-supported ultrathin Au nanowires: efficient electrocatalysts for borohydride oxidation†
†Electronic supplementary information (ESI) available. See DOI: 10.1039/c5cc06705g



**DOI:** 10.1039/c5cc06705g

**Published:** 2015-10-06

**Authors:** Annamalai Leelavathi, Rafia Ahmad, Abhishek K. Singh, Giridhar Madras, N. Ravishankar

**Affiliations:** a Centre for Nanoscience and Engineering , Indian Institute of Science , Bangalore-560012 , India; b Materials Research Centre , Indian Institute of Science , Bangalore-560012 , India . Email: nravi@mrc.iisc.ernet.in; c Department of Chemical Engineering , Indian Institute of Science , Bangalore-560012 , India

## Abstract

We report stable ultrathin Au nanowires supported on reduced graphene oxide with outstanding electrocatalytic activity for borohydride oxidation. Electrochemical impedance spectroscopy measurements showed abnormal inductive behavior, indicative of surface reactivation. DFT calculations indicate that the origin of the high activity stems from the position of the Au d-band center.

## Abstract 2

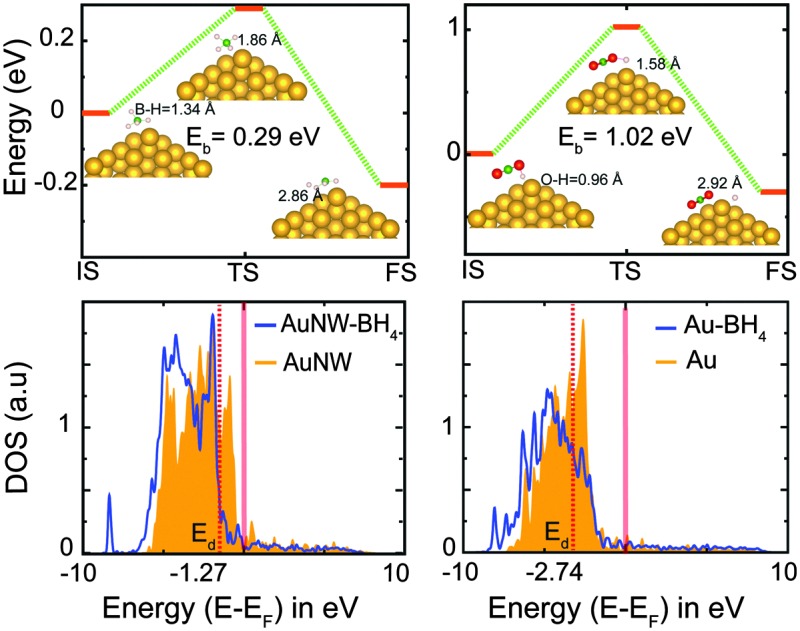
We report stable ultrathin Au nanowires supported on reduced graphene oxide with outstanding electrocatalytic activity for borohydride oxidation.

Rapidly dwindling fossil fuel reserves and increasing energy demands have provided an impetus to search for non-renewable resources among which fuel cells play an important part. Since the use of sodium borohydride for fuel cell applications,^1^ there have been significant efforts directed towards the development of the direct borohydride fuel cell (DBFC)^2^ as a carbon-free energy source owing to its high hydrogen content and energy capacity.^3^ In addition, sodium borohydride is less toxic, safe, chemically stable and amenable to transport in dry form. Nevertheless, DBFCs have limitations in terms of the availability of suitable anode materials/catalysts because of the complex eight-electron oxidation mechanism that often results in lack of selectivity towards elementary reaction steps. Although a Pt-based anode has less over-potential, the competition between hydrolysis and oxidation of borohydride limits the efficiency of fuel cells.^2^ Therefore, it is important to develop an anode material with a good selectivity towards oxidation of borohydride and higher stability under alkaline conditions. The most successful anode materials developed so far are based on Au nanostructures that exhibit high selectivity towards the borohydride oxidation step.^4,5^ However, unsupported Au nanocatalysts often suffer from aggregation during fuel cell operation^6^ and the inevitable presence of surfactants that limit electron transfer^7^ is a key obstacle for effective use of these nanostructures for practical applications.

To circumvent the above-mentioned problems, a pre-requisite step is to immobilize Au nanostructures on a high surface area conducting support that prevents aggregation and also provides flexibility to remove the undesired surfactant. In contrast to other supports, the inherent high surface area and superior electrical characteristics of graphene make it a unique candidate for electrocatalytic applications.^8–12^ Remarkably, wet chemical synthesis of reduced graphite oxide provides an advantage to decorate graphene sheets with surface functional groups that inhibit aggregation.^13^ These functional groups are also chemically active to facilitate effective heterogeneous nucleation of metal nanoparticles.^14^

There is increasing evidence that 1-D metallic nanostructures could be superior electrocatalysts and could overcome the inherent problems associated with nanoparticle catalysts.^15^ Recent studies have demonstrated that ultrathin (<2 nm diameter) nanowires and their hybrids exhibit enhanced electrocatalytic activity.^16–18^ In this communication, we present a detailed study on room temperature reduced GO supported ultrathin Au nanowires *via in situ* functionalization of OA with some exciting electrochemical experimental results and theoretical calculations. The interconnected ultrathin nanowire network supported on conductive rGO shows high electrical conductivity and electrocatalytic activity that are significantly better than those of Au nanoparticles supported on GO. The electrochemical performance of nanowire hybrids for borohydride oxidation was evaluated by cyclic voltammetry (CV) and electrochemical impedance spectroscopy (EIS). Furthermore, elementary reaction steps involved in the borohydride oxidation were screened by nudged elastic band (NEB) calculations that reveal a low reaction barrier for the nanowire catalyst compared with bulk Au.

The strategy for the synthesis of the rGO/Au nanowire hybrid involves the following key steps. Graphite is oxidized by a reported Hummer's method to yield GO that does not disperse in hexane.^19^ With the addition of oleylamine (OA), GO disperses in hexane. HAuCl_4_ and triisopropylsilane (TIPS) are added to the suspension and allowed to stand for 6 h leading to the growth of Au nanowires on chemically modified and reduced GO.^20–22^ Finally the product is centrifuged, washed several times with an ethanol and hexane mixture and stored in hexane. To obtain nanoparticle hybrids with comparable loading and particle size, we sonicated the GO/Au nanowire hybrids for 5 minutes leading to disintegration of the wires into particles (Fig. S1, ESI†).

Fig. 1a displays a low-magnification scanning electron microscopy (SEM) image of Au nanowires grown on GO sheets. The wires are uniformly distributed on the surface and span several microns in length (Fig. S2, ESI†). Bright-field TEM images reveal that the wires (∼2 nm diameter) (Fig. 1b) are well separated possibly due to the presence of the OA surfactant on their surface. The presence of wires only on the sheets or connected to the sheets suggests that there is strong anchoring of the wires on the GO substrate. As a consequence, the wires could be cleaned using polar solvents to remove the surfactant; similar cleaning of unsupported wires lead to their disintegration.^7^ The Au nanowires grown on GO also exhibited long-term stability; no aggregation was found even after storing the hybrid in ethanol for a month. This result emphasizes the crucial role of GO as a support in preserving the morphologies and size of ultrathin nanowires.

**Fig. 1:**
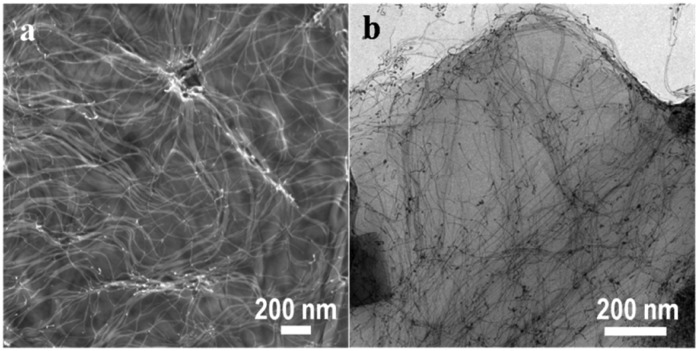
Micrographs of rGO/Au nanowires. (a) SEM images displaying the uniform growth of high aspect ratio ultrathin Au nanowires throughout the rGO sheet without any evident aggregation. (b) TEM image demonstrating the non-aggregated particle free nanowires.

We employed XPS for further characterization of fabricated nanowire hybrids. In addition to the presence of the Au(0) state (Fig. S3, ESI†), we note that the GO sheets are functionalized and have undergone reduction (Fig. S4, ESI†).^18^ The C 1s spectra of GO (Fig. S4a, ESI†) can be deconvoluted into five peaks at 284.5, 285.4, 286.5, 287.7 and 288.8 eV attributed to sp^2^C, sp^3^C or C–OH, epoxy C–O, carbonyl –C=O and carboxylate –COO– respectively.^23^ Correspondingly, the C/O ratio was calculated by measuring the peak area of C 1s (286.5 eV) to O 1s (532 eV) and was found to be 0.7 which confirms the oxidation of graphite. After keeping GO for 6 h in the Au nanowire growth solution, the C/O ratio increased to 21 accompanied by a decrease in the oxygenated carbon species (Fig. S4b, ESI†) indicating reduction of GO. This ratio compares well with the literature reported ratio for reduced GO under various harsh conditions.^24^ In addition, N 1s spectra (Fig. S5, ESI†) display two peaks at 398.8 and 400.8 eV, possibly due to amide and amine which confirms the chemical modification of GO.^25^ The observation of the additional peak at 285.5 eV corresponds to C–N further reinforcing the functionalization of GO by amine (Fig. S4b, ESI†).^26^

The reduction of GO is interesting as the process that we use for the formation of Au nanowires involves relatively mild conditions and room temperature that we do not associate with the harsher conditions required for GO reduction.^24^ To delineate the role of the different reagents used in the reduction of GO, we carried out several control experiments. As expected (Fig. S4c, ESI†), we note that the reduction proceeds even when the Au salt is not present in the solution indicating that either TIPS or OA is involved in the reduction process. The reduction proceeds when only OA is present in the solution while there is no reduction when only TIPS is present (Fig. S4d, ESI†), unambiguously pointing to the key role of OA in the reduction of GO. To support this premise, we monitored the C/O ratio of GO treated with OA in hexane solvent; with an increase of reaction time, the C/O ratio gradually increased to 1.8 and 2.9 for 6 and 30 h, respectively, as shown in Fig. S6 (ESI†). The progressive reduction of GO occurred due to deoxygenation indicating a nucleophilic interaction between OA and the epoxy group.^27^ The reduction process is also reflected in XRD^28^ (Fig. S7, ESI†), FTIR^19^ (Fig. S8, ESI†) and Raman^29^ (Fig. S9, ESI†) spectra.

The electrical conductivity of rGO/Au nanowires is several orders higher than that of rGO/Au nanoparticles (Fig. S10, ESI†). This is possibly due to the presence of a percolated network of nanowires on rGO. Such an increase in the conductivity is expected to enhance the electrocatalytic behavior of the rGO/Au nanowire hybrids. We investigated the nanowire hybrid as an anode for borohydride oxidation (BOX). Fig. 2a represents a comparison of BOX oxidation using rGO/Au nanowires and rGO/Au nanoparticle hybrids in 3 M NaOH containing 0.1 M of NaBH_4_ at a scan rate of 20 mV s^–1^.^30^ The electrochemical surface area (ECSA) was calculated from the reduction peak area (of the formed gold oxide) from cyclic voltammograms in a 3 M NaOH electrolyte. The BOX current was normalized with the respective ECSA for comparison of the activities. It should be noted that borohydride oxidation is a multi-step process; this is reflected in the voltammograms as a number of oxidation peaks. It has been reported that BOX proceeds *via* complex elementary reactions like B–H bond cleavage and B–O formation through intermediates.^31^ We obtained almost similar features with both the hybrids during CV scan with the nanowire hybrids exhibiting a significantly higher oxidation current than that of nanoparticle hybrids over the complete potential range due to less defects and easy diffusion of electrochemical species in the case of nanowires.^17^

**Fig. 2:**
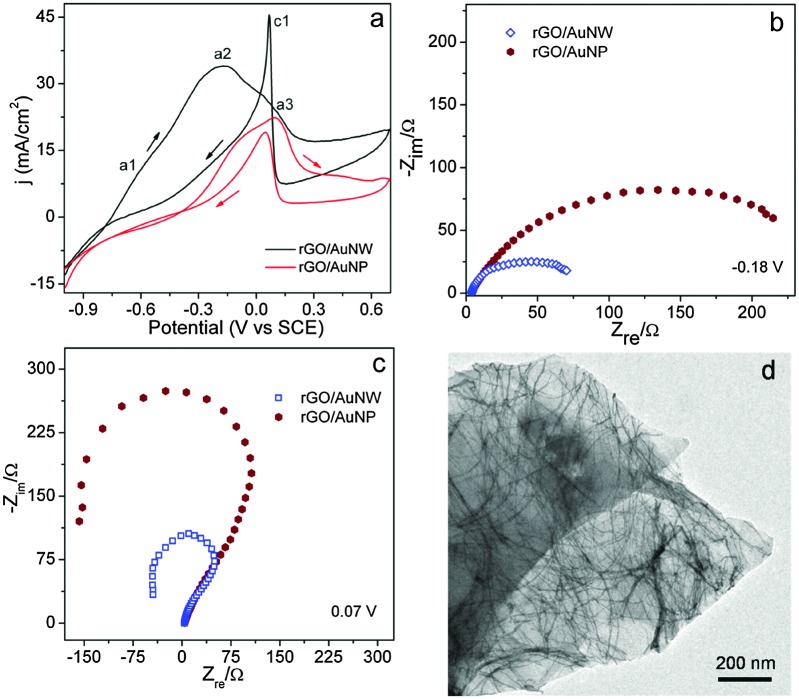
(a) CVs of synthesized hybrids in 3 M NaOH containing 0.1 M of NaBH_4_ at a scan rate of 20 mV s^–1^ clearly showing that the nanowire hybrids exhibit higher activity. Electrochemical impedance spectra measured in 3 M NaOH containing 0.1 M of NaBH_4_. (b) Nyquist plot at –0.18 V validating that the charge transfer resistance is less for nanowire than for nanoparticle hybrids. (c) Nyquist plot collected at 0.07 V showing negative Faradic impedance, which implies oxidative removal of BH_3_OH* intermediate species suggesting the transition of the Au electrode from a passive to an active state. (d) TEM micrograph of the rGO/Au nanowire recorded after BOX, indicating that the nanowires are stable during reaction.

The key reactions that take place during electrocatalytic oxidation of borohydride are listed here.

1 BH_4_^–^ + H_2_O → BH_3_OH^–^ + H_2_

2 BH_4_ + 8OH^–^ → BO_2_^–^ + 6H_2_O + 8e^–^

3 BH_3_* + OH^–^ → BH_3_OH* + e^–^

In the forward sweep (Fig. 2a), the small oxidation peak, a1 at –0.57 V *versus* SCE, is attributed to undesired hydrolysis of borohydride with evolution of H_2_ (reaction (1)).^32^ Unlike Pt, Au limits the Tafel (2H* → H_2_) and Volmer (H* + OH^–^ → H_2_O + e^–^) processes^33^ that are pre-requisites for the hydrolysis reaction.

The lower current at a1 implies that borohydride hydrolysis on Au wires is very limited. The peak a2 at –0.18 V corresponds to borohydride oxidation (reaction (2))^34^ which is an eight electron process. In this step, monoborane (BH_3_) is formed initially; BH_3_ can either engage with OH^–^ or dimerize to yield intermediates that are further oxidized to produce eight electrons. It should be noted that both reactions (1) and (3) result in the formation of BH_3_OH* as an intermediate.^34^ The shoulder peak a3 at 0.08 V is approximately the potential at which hydroxide species adsorb on the gold surface. The current density decreases with a further increase in potential due to the formation of Au oxide that may suppress the BOX reaction. The sharp peak (c1) that appears in the reverse scan is presumably due to removal of BH_3_OH* intermediates that are adsorbed on the electrode surface.^34^ This is key as it indirectly indicates recovery of Au active sites where further oxidation of borohydride takes place in the subsequent cycles. This is further verified by the appearance of inductive loops in the impedance spectra.

Fig. 2b and c depict the Nyquist plot of nanowire and nanoparticle rGO hybrids in 3 M NaOH containing 0.1 M NaBH_4_ at two different potentials. As expected, the resistance of nanowire hybrids is lower as compared to that of the nanoparticle hybrids; this observation is also consistent with higher currents in the case of the nanowire hybrids. The appearance of negative Faradic impedance at 0.07 V (Fig. 2c) implies that the rate-determining step for the reaction changes. Similar transitions from first to second quadrants have also been observed in alcohol oxidation on different electrodes^35^ suggesting the presence of inductive components involving in oxidative removal of intermediate poisoning species such as CO. In our case, the negative Nyquist plot can be explained by the removal of BH_3_OH* intermediate species suggesting the transition of the Au electrode from a passive to an active state.^36^ This is also reflected in the CV, as a sharp anodic peak in the reverse scan. Thus, oxidative removal of BH_3_OH* activates the Au sites for subsequent borohydride oxidation. This activation step is more pronounced in the case of nanowire hybrids. We also find that the nanowires are stable under the electrochemical conditions used and no significant changes were observed in the morphology or size of the wire after catalysis (Fig. 2d).

We performed density functional theory (DFT) calculations to obtain an insight into the observed higher catalytic activity of borohydride oxidation. Our calculations aim to simulate the barrier profile of reaction (2), which is considered as a key reaction for BOX. The structures of ultrathin Au nanowires were constructed by cleaving the bulk Au with low index surfaces. Our previous studies validated the success of this model to interpret the sensitivities of chemical analytes.^37^ On the other hand the bulk Au(111) surface was modelled. The calculated DFT adsorption energies showed that BH_4_ is more stable on nanowires than on the bulk. As seen in Fig. S11a (ESI†), the BH_4_ adsorption energy (*E*_ad_) on nanowires is –2.65 eV. Strongly adsorbed BH_4_ is distorted on the edge site of the nanowire (Fig. S11b, ESI†) and hence likely to be oxidized easily.^38^ The calculated BH_4_ *E*_ad_ of –1.72 eV on Au(111) is in good agreement with the existing literature.^31^ Evidently, BH_4_ adsorbs much strongly on the Au nanowire than on the bulk, which is indicative of better oxidation catalytic activity than that of the latter.^39^ To further confirm this, we perform reaction kinetics study through NEB calculations for complex steps involved in borohydride oxidation such as B–H cleavage and BO_2_ formation. The reaction profile involving initial, transition and final steps is shown in Fig. 3. B–H dissociation (Fig. 3a) and BO_2_ formation (Fig. 3b) energies are relatively lower for nanowires (0.29 eV and 1.02 eV) than for the bulk (Fig. S12, ESI,† 0.37 eV and 1.40 eV). This clearly validates that the rate limiting reaction barrier of BO_2_ formation is much lower on nanowires.

**Fig. 3:**
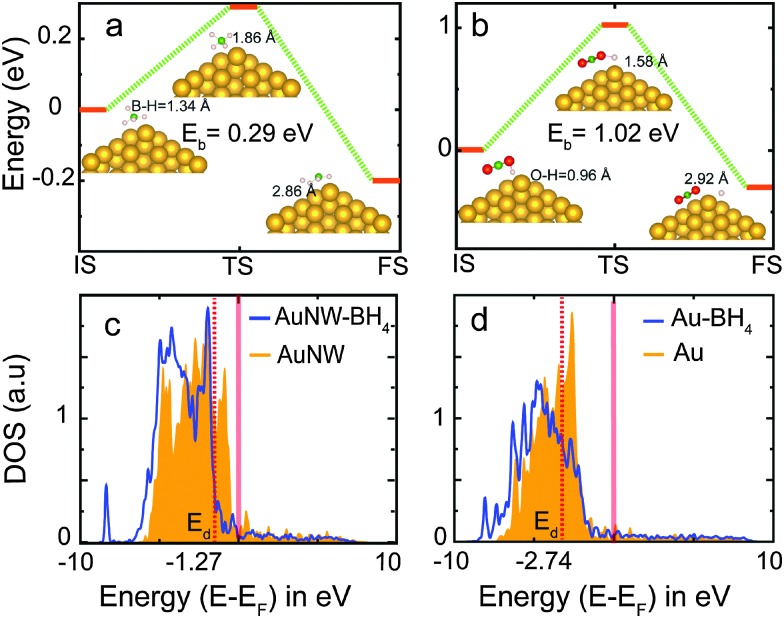
(a) BH_4_ dissociation: the reaction profile of BH_4_ dissociation on Au nanowires (NW). The transition state shows the exothermic reaction barrier. (b) BO_2_ formation: the reaction profile of BO_2_ formation on Au nanowires. The transition state (TS) shows the exothermic reaction barrier. IS, TS, and FS depict initial, transition, and final steps involved in the reaction. Gold, green, red, and pink atoms denote Au, B, O and H, respectively. Partial density of states for the d-orbital of (c) Au nanowires and (d) Au bulk (111). The red translucent line depicts the Fermi-level and the red dashed line is the indicator of the d-band center position. Shift of the DOS towards lower energy signifies the binding strength of the adsorbent in the case of nanowires.

In order to comprehend the different adsorption strengths of BH_4_ on the Au bulk and nanowires, we performed electronic structure analysis. The d-band model introduced by Hammer and Norskov provides an efficient tool to analyze the binding strength of adsorbents on transition metals.^40^ The position of the d-band center relative to the Fermi level is an indicator of the number of available antibonding states for the adsorbent. Fig. 3c and d show the position of the d-band center (red-dashed line) as –1.27 eV and –2.74 eV for the Au nanowire and bulk, respectively. Therefore, the number of empty antibonding d-states is higher in the case of a nanowire than bulk, leading to the better binding of BH_4_ on the former. The partial density of state plot shows that for Au-nanowires (Fig. 3c) the shift in the d-states towards lower energy is more than that of the bulk (Fig. 3d) upon BH_4_ adsorption. The shift of the density of states (DOS) towards lower energy signifies the binding strength of the adsorbent. Therefore, the binding energy of BH_4_ on nanowires is found to be much greater than that on the bulk.

In conclusion, we have reported a one-pot, OA mediated room temperature strategy for converting GO to amine functionalized rGO. The modification of rGO promotes the growth of ultrafine Au nanowires to form a hybrid structure. The resulting rGO/Au nanowires possess high electrocatalytic performance toward borohydride oxidation with a lower onset potential and higher oxidation current compared to nanoparticles. DFT calculations revealed that a shift in the position of the d-band center was the origin of enhanced activity of nanowires. Our synthesis method for making hybrids based on graphene and ultrathin Au nanowires and the insights obtained can be extended to other functional hybrids for energy-based applications.

N. R. acknowledges financial support from the Thematic Unit of Excellence (TUE), Nanomission and the Swarnajayanti fellowship of the DST, Govt of India. A. K. S. acknowledges SERC and MRC, IISc, for the computational facilities and TUE, Nanomission, DST for funding.
