# Supplementary material for: Graphene-oxide-supported ultrathin Au nanowires: efficient electrocatalysts for borohydride oxidation
Source: Chem Commun (Camb). 2015 Oct 6;51(94):16856–9. doi: 10.1039/c5cc06705g (PMC4757964; doi:10.1039/c5cc06705g)
Supplement: Supplementary file 1 [file CC-051-C5CC06705G-s001.pdf]

Electronic Supplementary Material (ESI) for ChemComm.
This journal is © The Royal Society of Chemistry 2015

Electronic Supplementary Information

Graphene Oxide Supported Ultrathin Au Nanowires: Efficient Electrocatalyst for Borohydride Oxidation

Annamalai Leelavathi,[a] Rafia Ahmad,[b] Abhishek K. Singh[b] Giridhar Madras[c] and N.Ravishankar[b]*

[a]Centre for Nanoscience and Engineering, Indian Institute of Science, Bangalore-560012, India.

[b]Materials Research Centre, Indian Institute of Science, Bangalore-560012, India.

[c]Department of Chemical Engineering, Indian Institute of Science, Bangalore-560012, India.

Experimental Section

Preparation of graphite oxide.

GO was prepared by using Hummer's method. 1 g of graphite (supplied by Graphite India Limited, Bangalore, India) was mixed with 23 mL of ice cooled concentrated sulfuric acid. Then, followed by slow addition of $KMnO_4$ (3 g) and the resultant mixture was stirred for 30 min at 35 ^oC. The solution temperature was increased to 98 ^oC by addition of water (45 mL) and maintained at this temperature for 15 min. Finally, 10 mL of H_2O_2 (30 % w/v) was added to terminated the oxidation reaction. The obtained product was washed multiple times with 5% HCl solution to remove undesired sulfate and finally vacuum dried.

Synthesis of rGO/Au nanowire.

The hybrid was prepared by injecting a 100 µL of oleylamine (w/v) into a hexane mixture containing $HAuCl_4$, followed by addition of 150 µL of TIPS.[1] This solution was sonicated for a min at room temperature and sequentially 15 mg of graphite oxide was added. The resultant reaction mixture was again sonicated for a min and then left undisturbed for 6 h. The reaction was stopped by centrifugation at 2000 rpm for 3 min to remove the unreacted TIPS. The obtained precipitate was washed several times with mixture of ethanol and hexane (1:1) solvent and then dispersed in hexane.

Characterization.

C/O ratio were determined by X-ray photoelectron spectroscopy (XPS), using Axis Ultra with monochromatic source of Al kα radiation and presence of nitrogen species were also confirmed by XPS N 1s spectra. The microstructural information was collected by using scanning electron microscopy (FE-SEM, Carl Zeiss Ultra 55) and transmission electron microscopy (TEM, FEI Tecnai T20 S-twin operated at 200 kV). To collect TEM micrographs, samples were prepared on carbon coated Cu-grids by drop casting a synthesized sample dispersed in hexane solvent and followed by drying at room temperature. Room temperature Raman scattering measurements was performed on LabRAM HR with an excitation wavelength of 514 nm using CCD detector. Fourier transform infrared spectroscopy (FTIR) was carried out on PerkinElmer Frontier FTIR equipped with Universal attenuated total reflectance accessory (UATR) with a resolution 4 cm^{-1}. Powder XRD was performed for synthesized samples using Phillips X'pert diffractometer with Cu Kα radiation. The current-voltage (I-V) electrical measurements were taken at ambient condition using Keithley source meter (Model no: 2420).

Electrochemical measurements.

To fabricate working electrodes, synthesized samples were dispersed in hexane to make a catalyst ink and drop casted on graphite paper, followed by drying at room temperature. Cyclic voltammetry (CV) was carried out in a three electrode electrochemical cell by utilizing Pt and Calomel as counter and reference electrode, respectively. For all the measurements, 3 M NaOH was used as an electrolyte and performed at room temperature.

***Electrochemical Impedance Spectroscopy (EIS) Measurements*.**

EIS was recorded using a CHI 660D electrochemical workstation. The spectra were recorded at an AC signal of 10 mV from 100 kHz to 10 MHz.

***Computational methods*.**

The first principles calculations were performed using density functional theory (DFT) as implemented in the Vienna ab initio simulation package (VASP).[2] Electron-ion interactions were described using all-electron projector augmented wave (PAW) pseudopotentials.[3, 4] Electronic exchange and correlation were approximated by a Perdew-Burke-Ernzerh of generalized gradient approximation. We modeled the wires with a hexagonal prism morphology bounded by {110} planes with the long axis along [111] direction. All integrations over the one-dimensional irreducible Brillouin zone were performed by using a Monkhorst−Pack 1 × 1 × 5 k-grid. The dimension of the unit cell along the longitudinal direction (periodic direction) was fully optimized and found to be 7.17 Å. For the adsorption and oxidation calculations, we considered a supercell of 1 × 1 × 2, to ensure a vacuum space of 12 Å between the periodic replicas to avoid any spurious interactions. The calculations on nanoparticles modeled as Au (111) surface were carried out in a well converged k-mesh of 7 x 7 x 1. A conjugate gradient scheme is used to relax the structures until the component of the forces on each atom was <0.005 eV $Å^{-1}$. The cut-off energy was set to 400 eV to ensure the accuracy of the results. The minimization of the reaction pathways and the search of the transition states (TS) have been performed with the steepest-descent Nudged Elastic Band method (NEB).[5, 6] The reaction pathways and barriers were then calculated by NEB method through the identified TS.

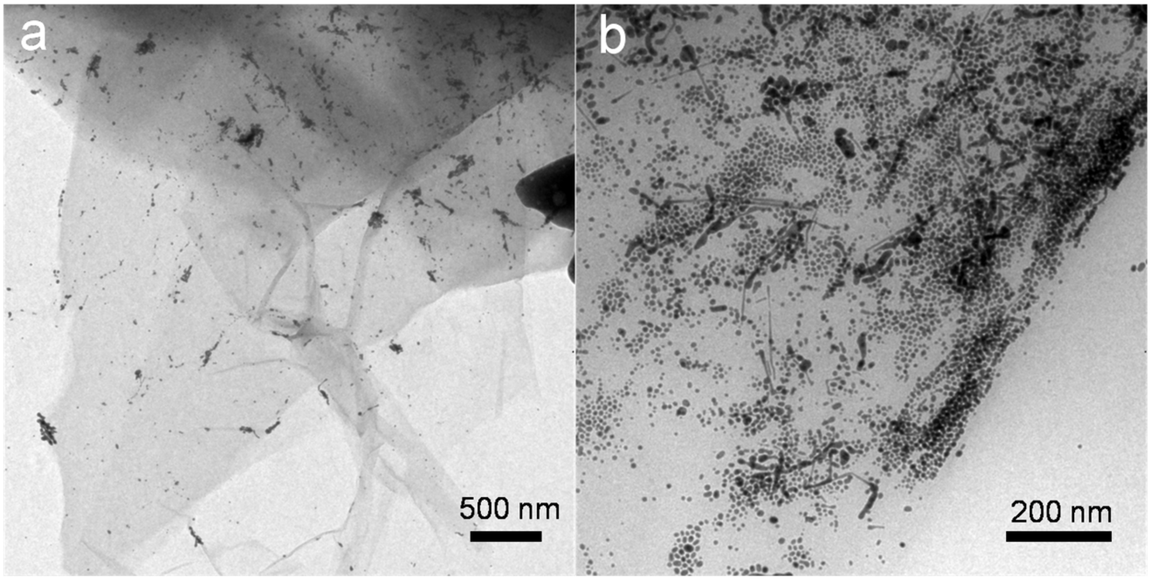

Fig. S1 (a & b) Bright field TEM micrographs of rGO/Au nanoparticle at different magnifications. rGO/Au nanowire hybrids was employed as precursors and sonicated for 5 minutes to disintegrate wires into particles.

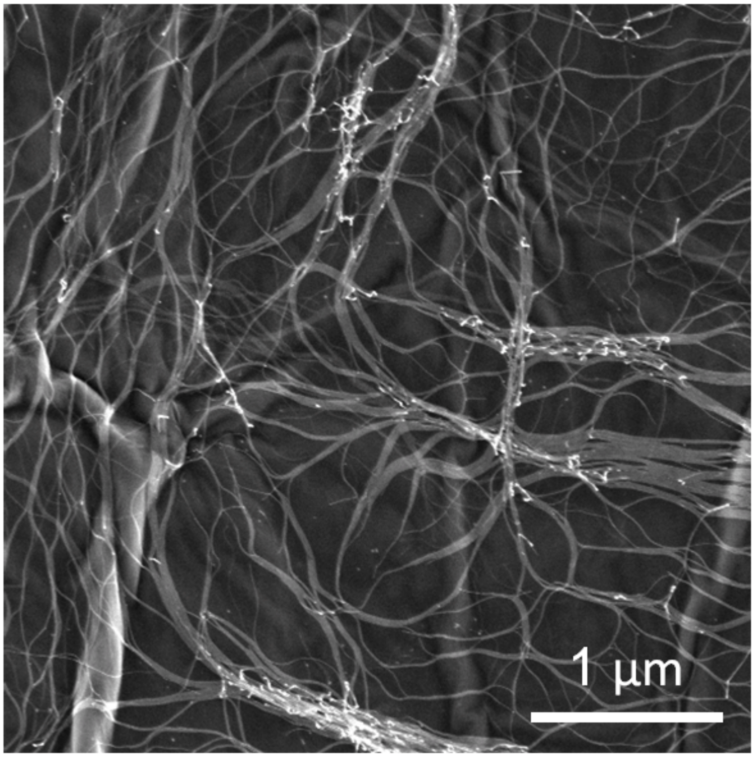

Fig. S2 Low magnification SEM image display the uniform growth of high aspect ratio ultrathin Au nanowires throughout the rGO sheet.

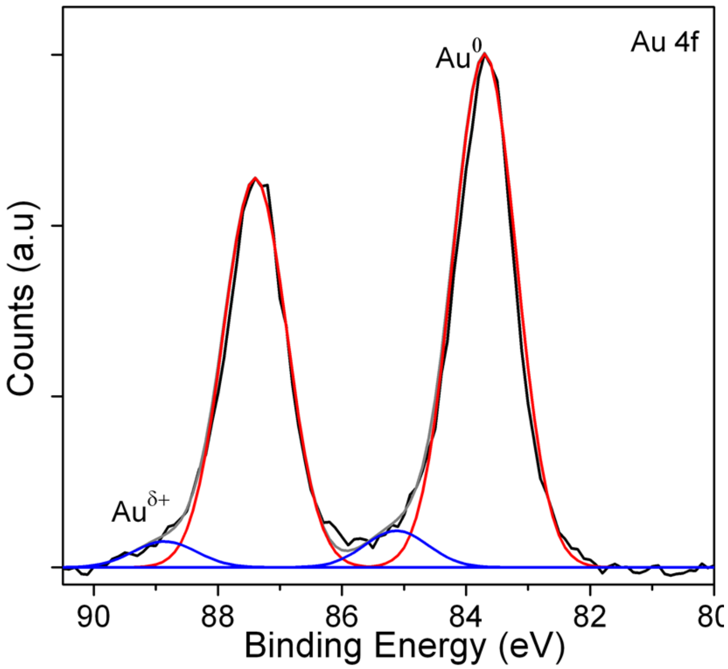

Fig. S3 XPS Au 4f spectra of rGO/Au nanowire, confirms the presence of Au^0 state.

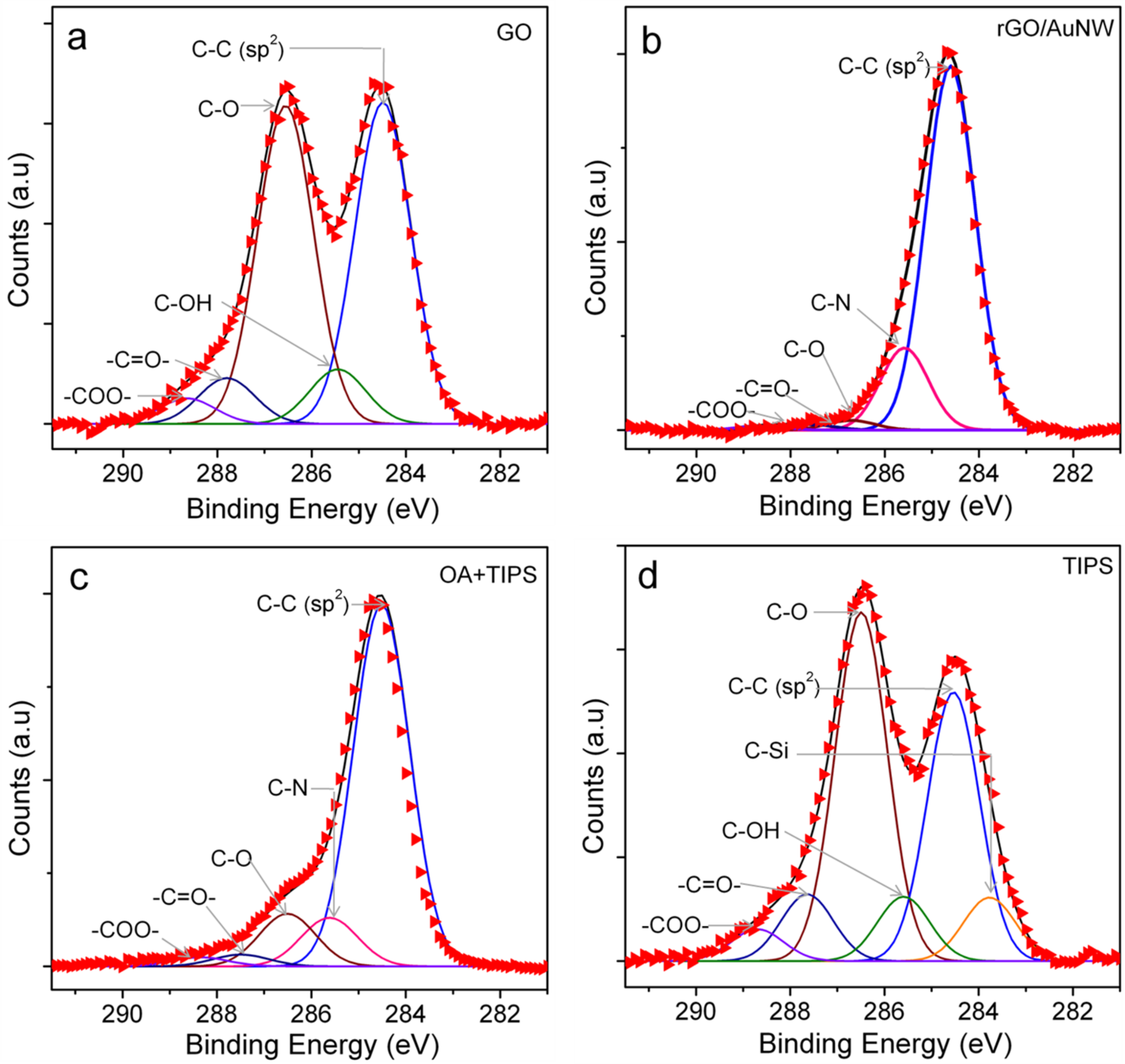

Fig. S4 C 1s spectra of (a) GO shows the presence of oxygenated species. (b) rGO/Au nanowire (NW) (GO was added to the hexane solution containing nanowire growth solution consist of HAuCl4, Oleylamine (OA) and triisopropylsilane (TIPS)). It is observed that C/O ratio increased, signifying that reduction of GO had happened. Appearance of new peak at 285.4 eV, suggesting chemical modification of GO and hence a chemical bond between carbon and nitrogen.(c) GO reduction happened even when the Au salt is not present in the solution (with OA+TIPS). (d) GO treated with TIPS, almost no change in C/O ratio. Unambiguously pointing to the key role of OA in the reduction of GO. OA addition leads to reduction and functionalization of GO.

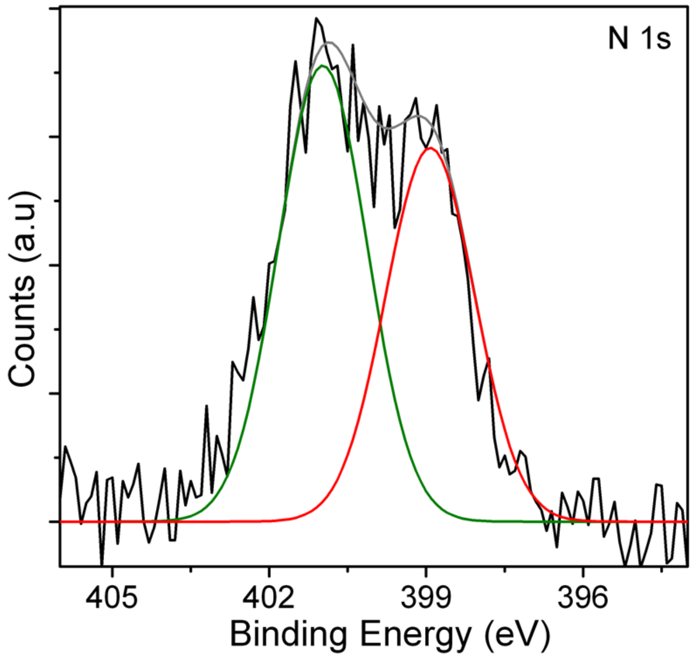

Fig. S5 Deconvoluted XPS N 1S of rGO/Au nanowire shows two peaks probably due to presence of oleylamine and oleylammonium cation,[7] which confirms the successful grafting of OA on GO.

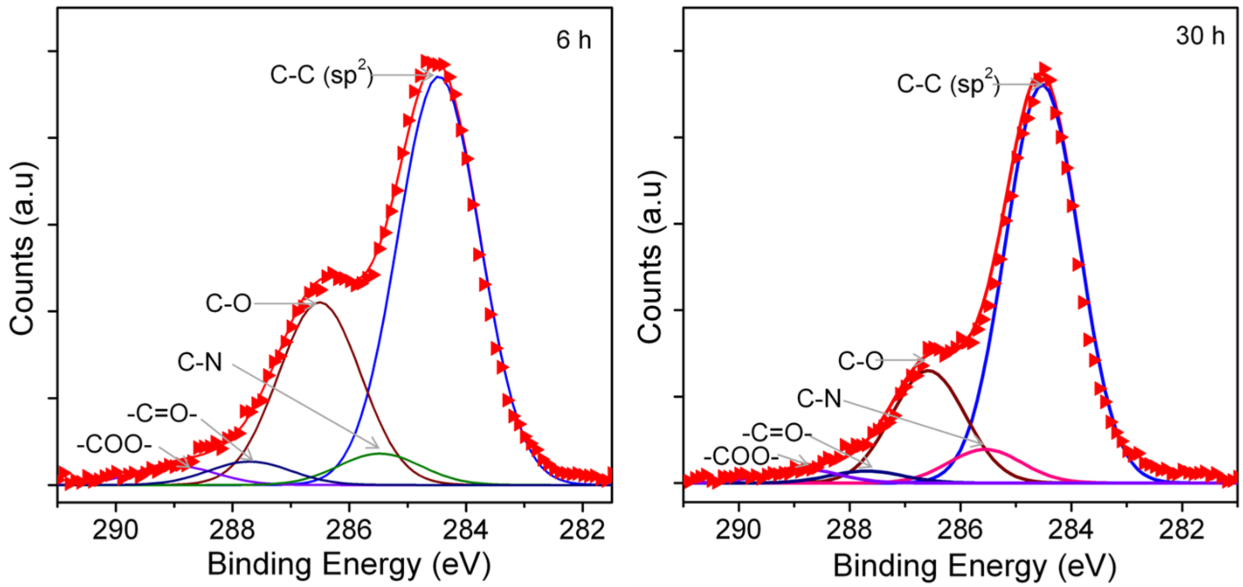

Fig. S6 XPS measurements for GO samples treated with OA (in hexane solution at room temperature) showing deoxygenation, and with decrease in epoxy groups. Legend: 6 h and 30 h represent GO reduction in the presence of OA as a function of reaction time 6 and 30 h.

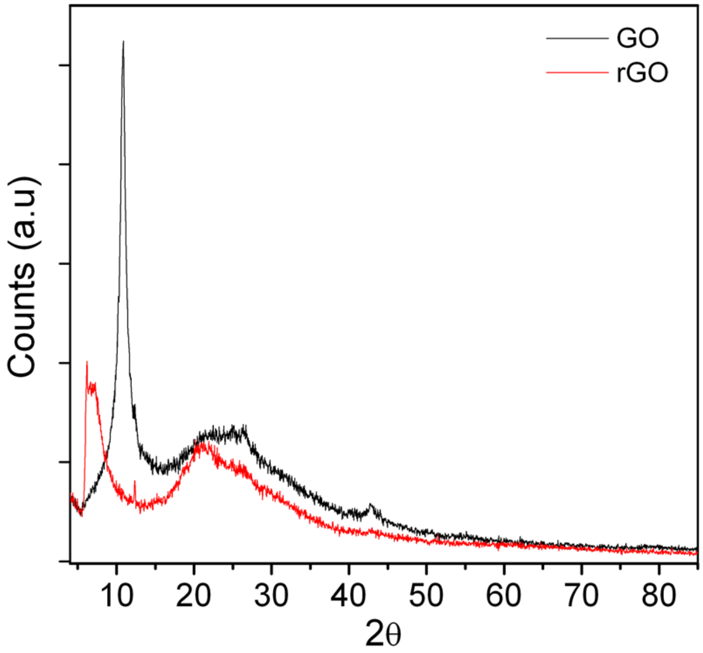

Fig. S7 XRD pattern of GO before and after addition of oleylamine (OA), the peak that appears around 6.8 (2θ) is ascribed to intercalation of OA on rGO. The d-spacing increase after OA treatment confirms the room temperature functionalization of GO.

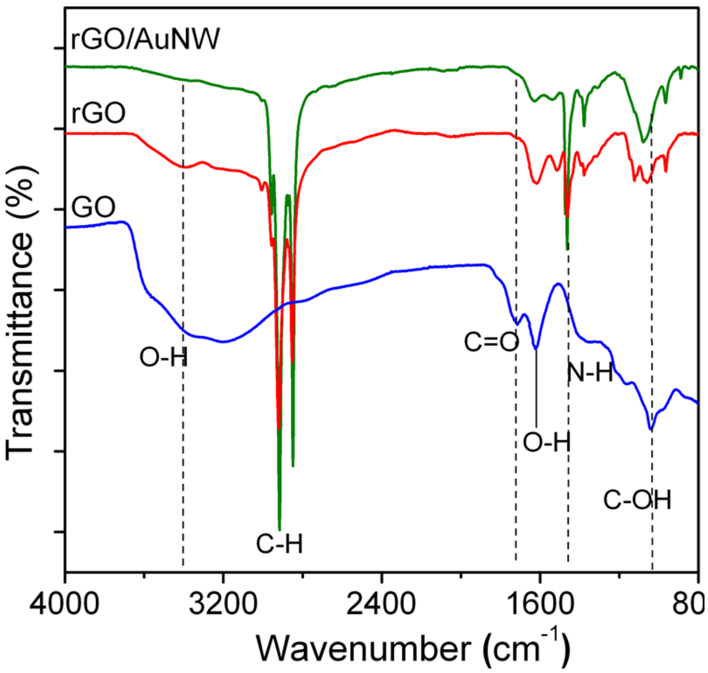

Fig. S8 FTIR of synthesized samples illustrates that the stretching vibration bands of hydroxyl, epoxy and carboxyl decreased with addition of OA to GO suspension, thus indicates the reduction of GO and also deoxygenation process.[8] In addition appearance of N-H validates the grafting of amine on sheets.

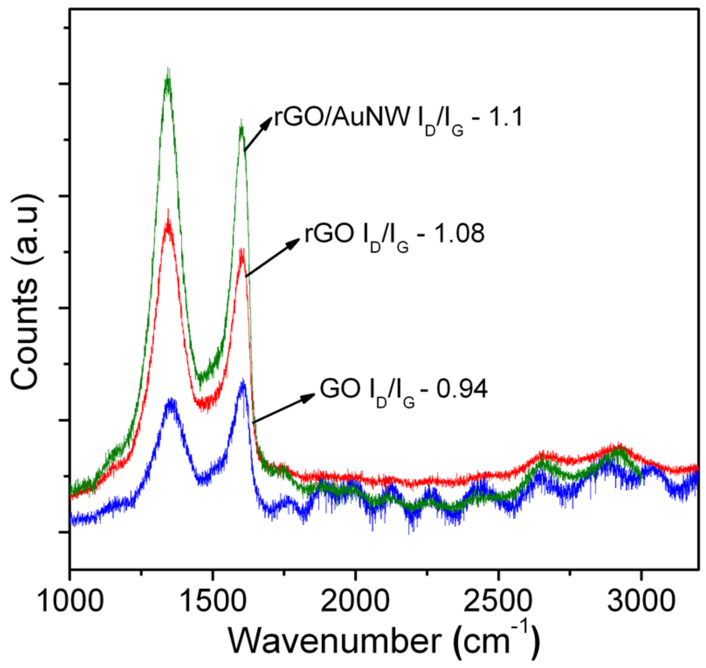

Fig. S9 Raman spectra shows well documented two peaks around 1342 and 1600 cm^{-1},[9] corresponds to D and G band, respectively. Compared with GO, the I_D/I_G ratio for rGO and rGO/Au nanowires increased,[10] such intensity increase could be due to sp^2 domain size reduction and also describes the newly formed domains which are smaller in size.[11]

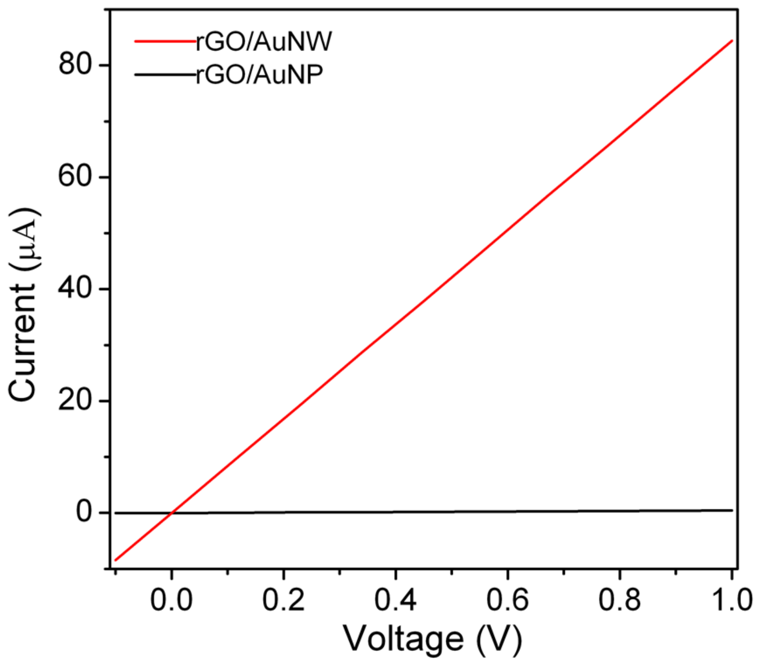

Fig. S10 I-V measurements of synthesized hybrids. Electrical conductivity of rGO/AuNW was several orders higher than that of rGO/AuNP might be due to formation of low percolation threshold which originates from high aspect ratio wires.[12]

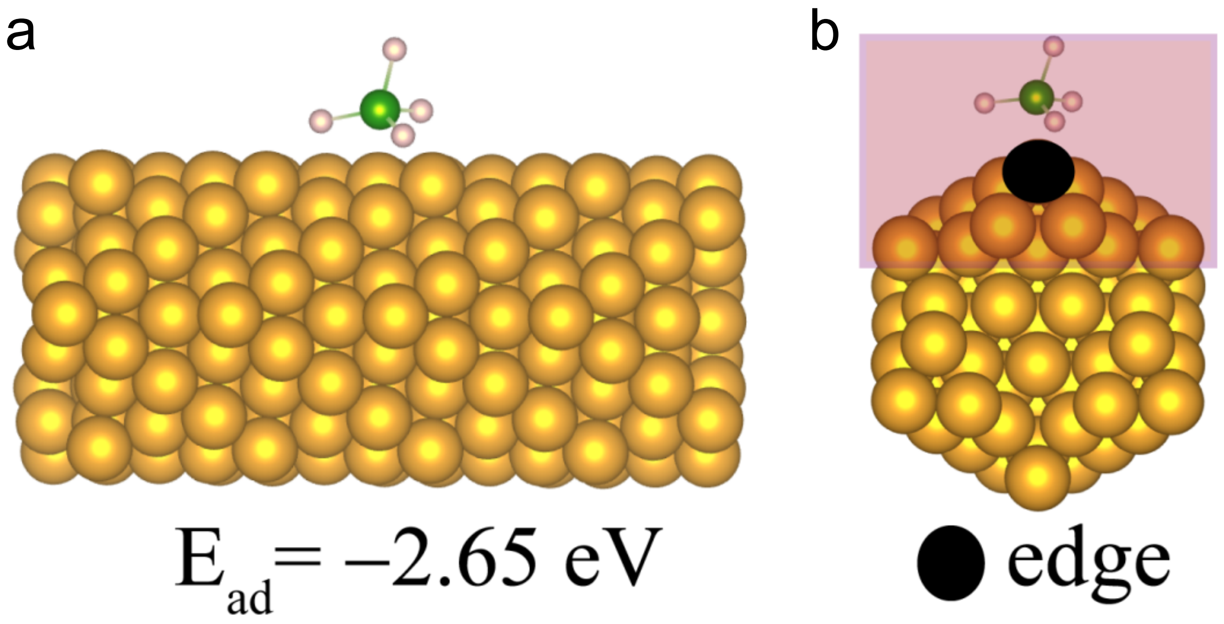

Fig. S11 (a) The side and cross-sectional view of the BH_4 adsorbed on Au nanowire, adsorption energy (E_{ad}) on nanowire is -2.65 eV (b) Strongly adsorbed BH_4 is distorted on the edge site of the nanowire, it is likely to exhibit good BH_4 oxidation catalytic activity. Gold, green, and pink atoms denote Au, B and H, respectively.

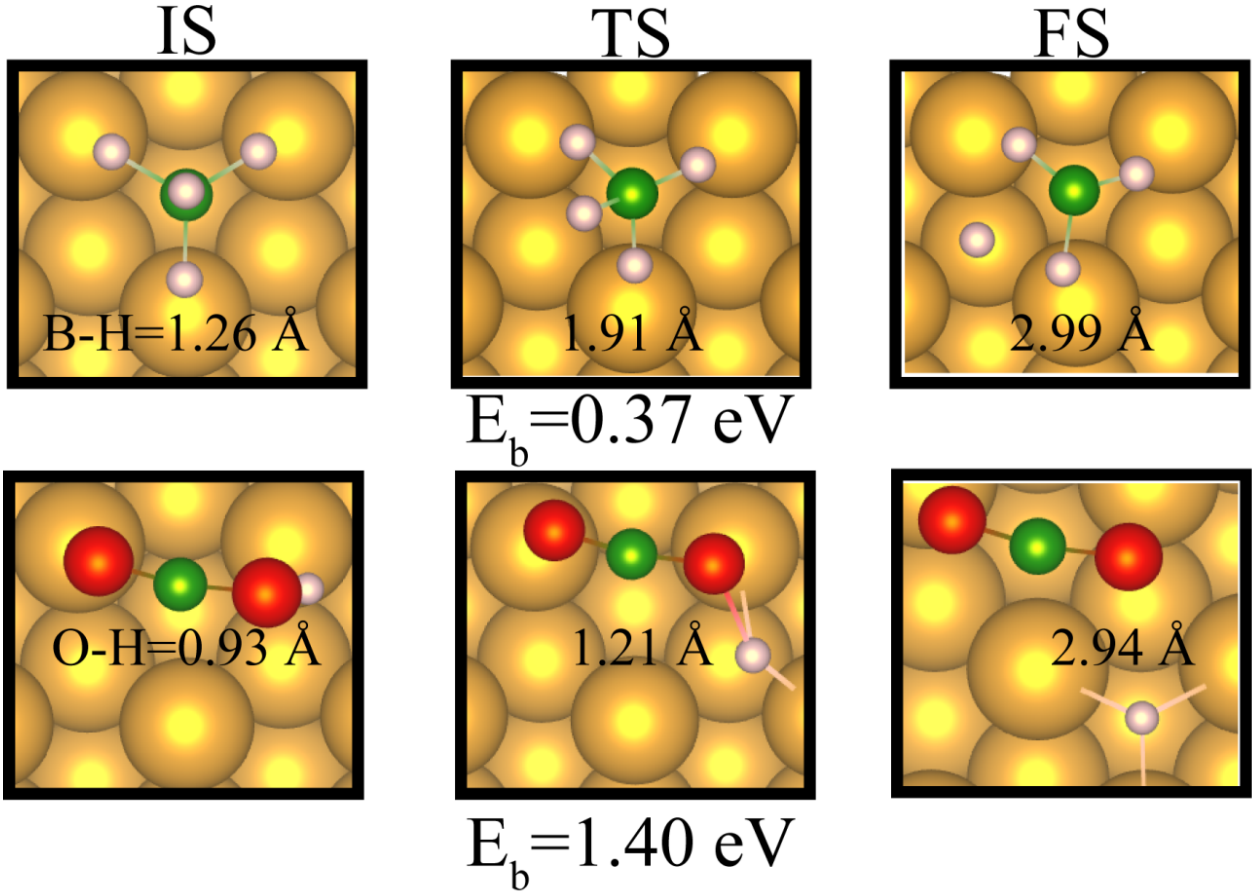

Fig. S12 The reaction profile involving initial, transition and final steps for B-H dissociation and BO_2 formation energies (E_b) on Au (111) are shown here. E_b for dissociation and BO_2 formation energies are 0.37 eV and 1.40 eV, respectively which are relatively higher for bulk when compared with nanowire hybrid (Figure 4). Gold, green, red, and pink atoms denote Au, B, O and H, respectively.

References

1. Feng, H.; Yang, Y.; You, Y.; Li, G.; Guo, J.; Yu, T.; Shen, Z.; Wu, T.; Xing, B. *Chem. Commun.* **2009**, 1984-1986.
2. Kresse, G.; Hafner, J. *Phys. Rev. B* **1993,** *47*, 558.
3. Kresse, G.; Joubert, D. *Phys. Rev. B* **1999,** *59*, 1758.
4. Blöchl, P. E. *Phys. Rev. B* **1994,** *50*, 17953.
5. Sheppard, D.; Terrell, R.; Henkelman, G. *J. Chem. Phys.* **2008,** *128*, 134106.
6. Henkelman, G.; Arnaldsson, A.; Jónsson, H. *J. Chem. Phys.* **2006,** *124*, 044706.
7. Yang, K.; Liang, S.; Zou, L.; Huang, L.; Park, C.; Zhu, L.; Fang, J.; Fu, Q.; Wang, H. *Langmuir* **2012,** *28*, 2904-2908.
8. Nethravathi, C.; Rajamathi, M. *Carbon* **2008,** *46*, 1994-1998.
9. Jasuja, K.; Berry, V. *ACS Nano* **2009,** *3*, 2358-2366.
10. Peng, S.; Li, L.; Han, X.; Sun, W.; Srinivasan, M.; Mhaisalkar, S. G.; Cheng, F.; Yan, Q.; Chen, J.; Ramakrishna, S. *Angew. Chem. Int. Ed.* **2014,** *53*, 12594-12599.
11. Ferrari, A.; Robertson, J. *Phys. Rev. B* **2000,** *61*, 14095.
12. Madaria, A. R.; Kumar, A.; Ishikawa, F. N.; Zhou, C. *Nano Res.* **2010,** *3*, 564-573.
